# Supplementary figures and images for: Comparative Phosphoproteomic Analysis of Sporulated Oocysts and Tachyzoites of Toxoplasma gondii Reveals Stage-Specific Patterns
Source: Molecules. 2022 Feb 2;27(3):1022. doi: 10.3390/molecules27031022 (PMC8839046; doi:10.3390/molecules27031022)

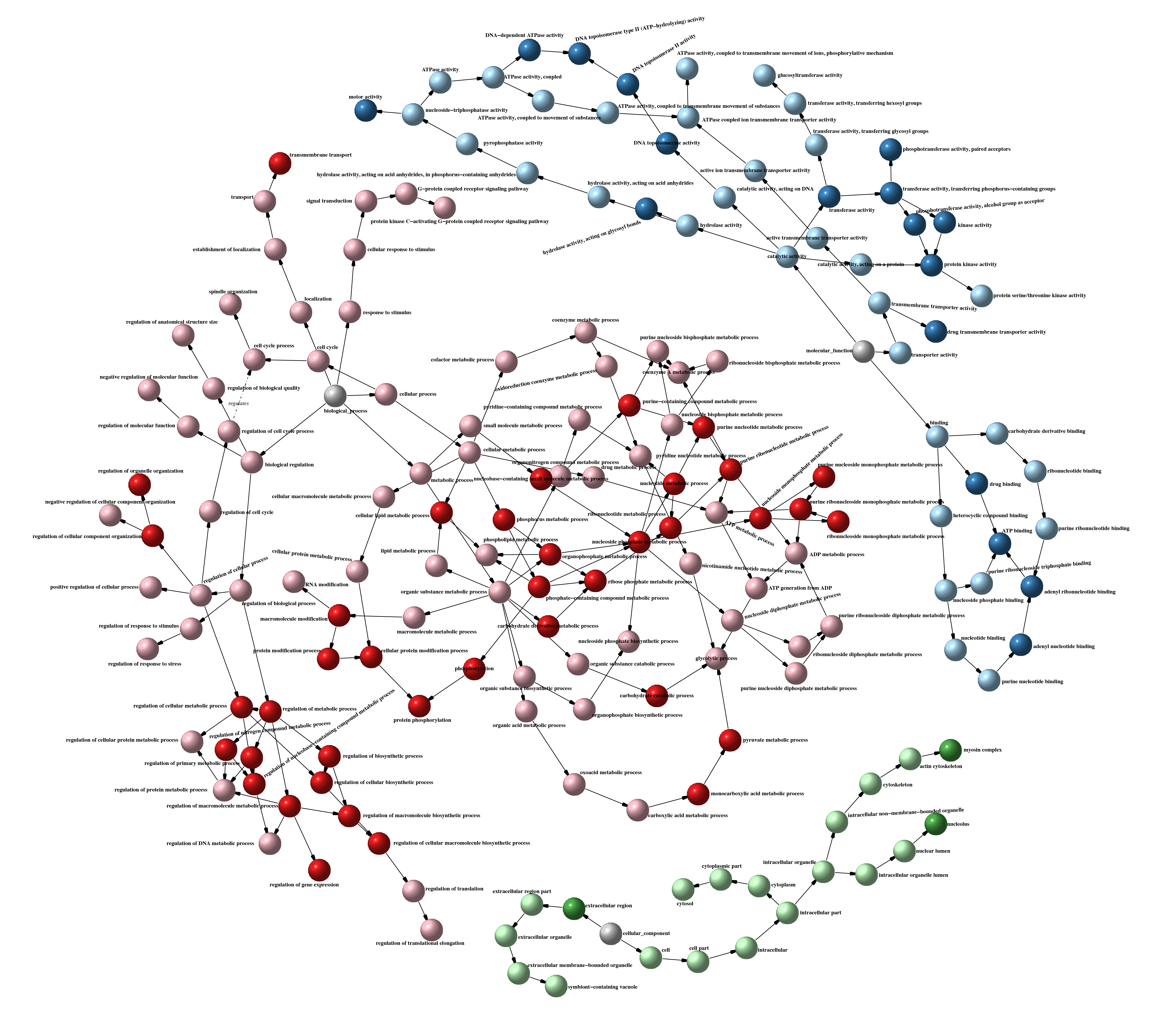

Supplement: Supplementary file 1 [file molecules-27-01022-s001.zip › Figure S1.tif]

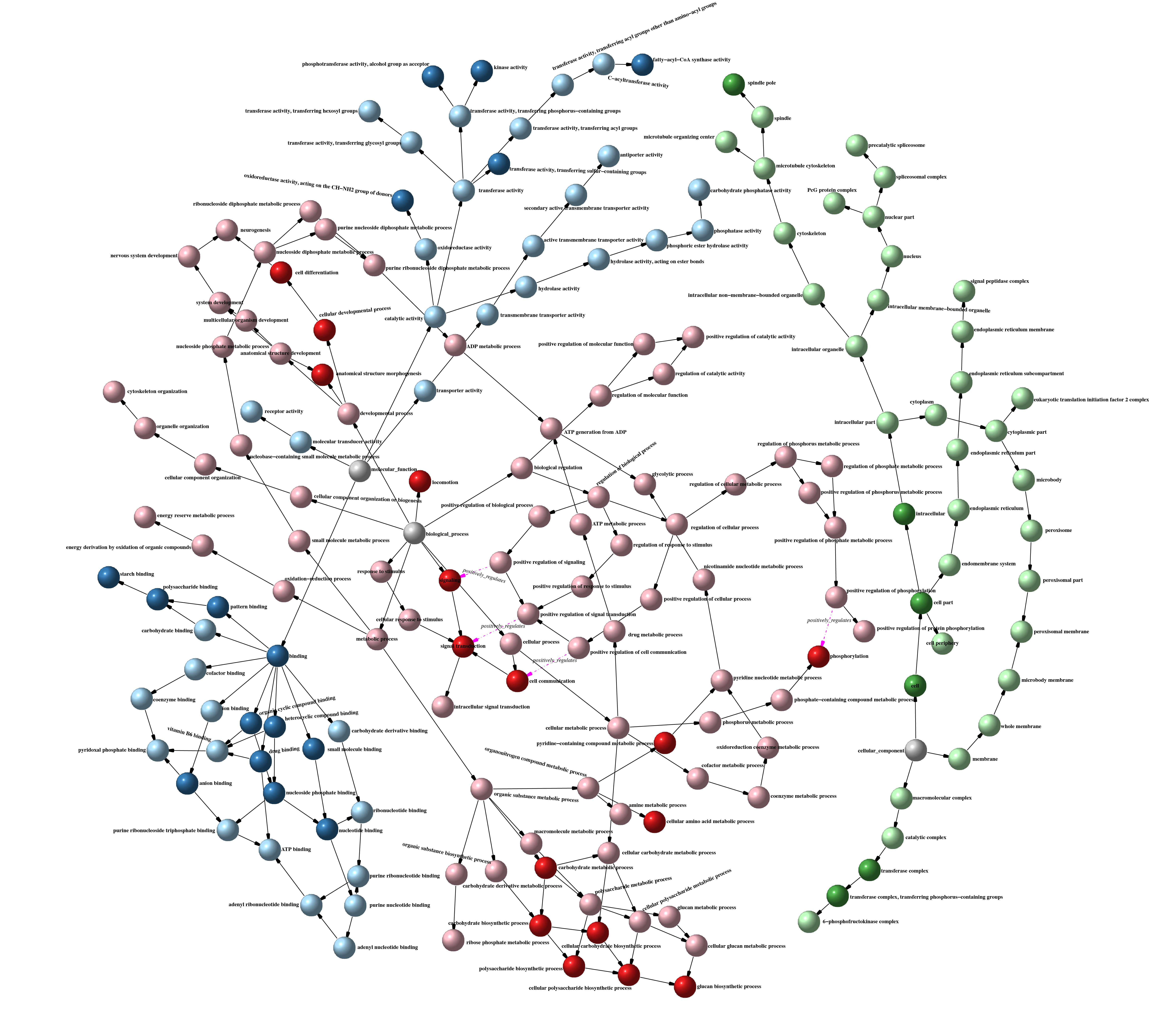

Supplement: Supplementary file 1 [file molecules-27-01022-s001.zip › Figure S2.tif]
